# Cardiac energetics, oxygenation, and perfusion during increased workload in patients with type 2 diabetes mellitus

**DOI:** 10.1093/eurheartj/ehv442

**Published:** 2015-09-20

**Authors:** Eylem Levelt, Christopher T. Rodgers, William T. Clarke, Masliza Mahmod, Rina Ariga, Jane M. Francis, Alexander Liu, Rohan S. Wijesurendra, Saira Dass, Nikant Sabharwal, Matthew D. Robson, Cameron J. Holloway, Oliver J. Rider, Kieran Clarke, Theodoros D. Karamitsos, Stefan Neubauer

**Affiliations:** 1 Division of Cardiovascular Medicine, Radcliffe Department of Medicine, University of Oxford Centre for Clinical Magnetic Resonance Research, University of Oxford, John Radcliffe Hospital, Headley Way, Oxford OX3 9DU, UK; 2 Department of Physiology, Anatomy, and Genetics, University of Oxford, Oxford, UK; 3 Oxford Heart Centre, John Radcliffe Hospital, Oxford, UK; 4 St. Vincent's Hospital, Sydney, Australia; 5 1st Department of Cardiology, AHEPA Hospital, Aristotle University, Thessaloniki, Greece

**Keywords:** Coronary microvascular function, Diabetes mellitus, Diabetic cardiomyopathy, Metabolism, Oxygen

## Abstract

**Aims** Patients with type 2 diabetes mellitus (T2DM) are known to have impaired resting myocardial energetics and impaired myocardial perfusion reserve, even in the absence of obstructive epicardial coronary artery disease (CAD). Whether or not the pre-existing energetic deficit is exacerbated by exercise, and whether the impaired myocardial perfusion causes deoxygenation and further energetic derangement during exercise stress, is uncertain.

**Methods and results** Thirty-one T2DM patients, on oral antidiabetic therapies with a mean HBA1c of 7.4 ± 1.3%, and 17 matched controls underwent adenosine stress cardiovascular magnetic resonance for assessment of perfusion [myocardial perfusion reserve index (MPRI)] and oxygenation [blood-oxygen level-dependent (BOLD) signal intensity change (SIΔ)]. Cardiac phosphorus-MR spectroscopy was performed at rest and during leg exercise. Significant CAD (>50% coronary stenosis) was excluded in all patients by coronary computed tomographic angiography. Resting phosphocreatine to ATP (PCr/ATP) was reduced by 17% in patients (1.74 ± 0.26, *P* = 0.001), compared with controls (2.07 ± 0.35); during exercise, there was a further 12% reduction in PCr/ATP (*P* = 0.005) in T2DM patients, but no change in controls. Myocardial perfusion and oxygenation were decreased in T2DM (MPRI 1.61 ± 0.43 vs. 2.11 ± 0.68 in controls, *P* = 0.002; BOLD SIΔ 7.3 ± 7.8 vs. 17.1 ± 7.2% in controls, *P* < 0.001). Exercise PCr/ATP correlated with MPRI (*r* = 0.50, *P* = 0.001) and BOLD SIΔ (*r* = 0.32, *P* = 0.025), but there were no correlations between rest PCr/ATP and MPRI or BOLD SIΔ.

**Conclusion** The pre-existing energetic deficit in diabetic cardiomyopathy is exacerbated by exercise; stress PCr/ATP correlates with impaired perfusion and oxygenation. Our findings suggest that, in diabetes, coronary microvascular dysfunction exacerbates derangement of cardiac energetics under conditions of increased workload.

## Introduction

Diabetes mellitus (DM) is associated with increased risk of congestive heart failure^1^ and cardiovascular mortality.^2^ Myocardial energy depletion^3,4^ and coronary microvascular dysfunction^5^ are features of diabetic heart disease. Myocardial energy depletion in patients with diabetes is a multifactorial phenomenon, related to limitations in uptake and utilization of substrates,^6^ mitochondrial dysfunction,^7^ and impaired energy transfer from mitochondria to myofibrils.^8^ These metabolic changes, in combination with impaired myocardial perfusion, may decrease the ability of the diabetic heart to adapt to acute increases in workload. Further derangement of the energetic deficit on increased workload could potentially limit myocardial contractile reserve and exacerbate diastolic dysfunction and stimulate maladaptive pathways, eventually leading to heart failure.^9,10^

Phosphorus-magnetic resonance spectroscopy (^31^P-MRS) allows non-invasive assessment of the myocardial phosphocreatine to ATP concentration ratio (PCr/ATP), which is a sensitive indicator of the myocardial energy status.^11^ Using ^31^P-MRS, we, and others, have shown that the diabetic heart is energetically compromised, with a decreased PCr/ATP, at rest.^3,4^ However, changes in cardiac metabolic reserve and energy metabolism in diabetic patients under conditions of increased workload have not been studied.

Cardiovascular magnetic resonance (CMR) during the first pass of an injected tracer permits assessment of myocardial perfusion reserve during pharmacological stress.^12^ Abnormal perfusion reserve in the absence of a significant coronary stenosis is likely to reflect coronary microvascular dysfunction, although separation of the contribution from impaired vasodilation of epicardial muscular arteries and impaired vasodilation of arterioles is not yet possible based on these techniques.^13,14^ Furthermore, blood-oxygen level-dependent (BOLD) CMR or oxygenation-sensitive CMR has the ability to non-invasively assess myocardial tissue oxygenation during vasodilator stress, providing a more direct measure of microvascular dysfunction and ischaemia than perfusion.^15,16^ Oxygenation-sensitive CMR can non-invasively assess myocardial tissue oxygenation without the need for exogenous contrast by measuring BOLD signal intensity (SI) differences, which reflect deoxygenated haemoglobin concentration during adenosine stress.^15,17^ Although the technique has some limitations for widespread clinical use,^18^ the potential benefits of BOLD imaging were demonstrated in multiple clinical studies.^19–21^ Thus, CMR allows a comprehensive investigation of the interplay between metabolic and ischaemic changes in the diabetic heart.

The primary objective of this study was to assess whether the pre-existing cardiac energetic deficit is exacerbated by exercise in patients with type 2 diabetes mellitus (T2DM) as a measure of metabolic reserve. The second objective was to assess myocardial perfusion reserve and oxygenation during vasodilator stress and to examine their relationship with myocardial energy status in T2DM patients, who were free of significant epicardial coronary artery stenosis. We hypothesized that the intrinsic metabolic deficit and coronary microvascular dysfunction in diabetes, either alone or in combination, will reduce the ability of the diabetic myocardium to adapt to acute increases in workload and exacerbate the energetic derangement.

## Methods

### Subjects

The study complies with the Declaration of Helsinki and was approved by the National Research Ethics Committee (REC Ref. 13/SW/0257), and informed written consent was obtained from each participant. Thirty-nine subjects with T2DM on oral antidiabetic therapies and 17 volunteers of similar age and body mass index (BMI) were recruited. T2DM was diagnosed according to the World Health Organization criteria.^22^

### Inclusion and exclusion criteria

**Figure 1:**
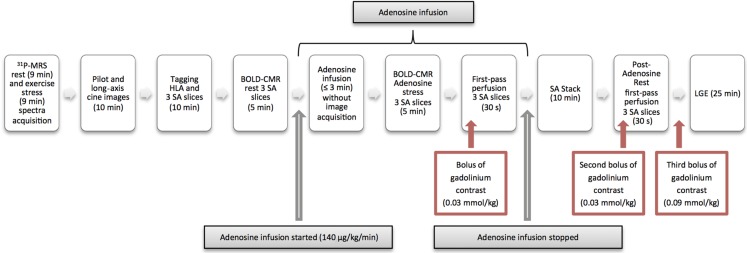
Timeline for scan protocol. Cardiac ^31^P-MRS (3 T) was performed first at rest (9 min) and then during 9 min of leg exercise lying prone, with 2.5 kg weights attached to both legs. This was followed by CMR scan (3 T). CMR included pilot and cine imaging to assess LV volumes, mass, and ejection fraction; myocardial tagging imaging at horizontal long axis; and three ventricular SA slices (basal, mid, and apical LV). For oxygenation-sensitive CMR (BOLD-CMR), three ventricular SA slices (basal, mid, and apical) were acquired at rest. Adenosine (140 µg/kg/min) was then infused for at least 3 min, and the same three BOLD images were acquired during stress. Subsequently, a 0.03 mmol/kg bolus of gadolinium-based contrast (Gadoterate meglumine, Dotarem, Guerbet LLC) was injected for first-pass perfusion imaging. Adenosine was then discontinued; after 10 min of break from scanning, SA stack images were obtained, with the heart rate returned to baseline rest measurements. After at least 20 min to allow adenosine and gadolinium contrast washout, another (second) 0.03 mmol/kg bolus of gadolinium was given for post-adenosine rest perfusion imaging. A third bolus of 0.09 mmol/kg gadolinium was then given for LGE to exclude fibrosis.

Subjects were excluded if they had a history of cardiovascular disease, chest pain, tobacco smoking, uncontrolled hypertension [resting systolic blood pressure (BP) >140 mmHg and diastolic BP >90 mmHg], contraindications to MR imaging (MRI), ischaemic changes on 12-lead ECG, or renal impairment (estimated glomerular filtration rate below 30 mL/min). T2DM participants were excluded if they were taking insulin. Additionally, patients were screened for obstructive epicardial CAD (>50% of luminal stenosis) by coronary computed tomographic angiography (CCTA). Subjects with no evidence of significant epicardial CAD on CCTA underwent CMR, ^31^P-MRS (*Figure 1*), transthoracic echocardiography, and fasting blood tests.

### Coronary computed tomographic angiography

CCTA scans were performed on a 64-slice CT scanner (Discovery 690, GE Healthcare, City, USA) in accordance with guidelines from the Society of Cardiovascular Computed Tomography.^23^ Participants received beta-blockade (intravenous metoprolol) and sublingual GTN to achieve a heart rate of <65 b.p.m. A preliminary unenhanced scan was performed to assess coronary artery calcium score. During the CCTA acquisition, 80 mL of iodinated contrast (Visipaque, GE Healthcare, Princeton, NJ, USA) was injected followed by a 50 mL saline flush. Significant coronary artery disease (CAD) was defined as >50% luminal stenosis.

### Cardiac magnetic resonance protocol

CMR was performed on a 3 T system (TIM Trio; Siemens Healthcare). All participants refrained from caffeine ingestion for 24 h and were scanned after fasting overnight. Cine imaging was performed using standard methods.^24^ Strain imaging was performed using myocardial tagging sequence, as described previously.^25^

Oxygenation-sensitive CMR and stress perfusion CMR were performed as described previously.^21,26^ For oxygenation-sensitive CMR, three ventricular short-axis (SA) slices (basal, mid, and apical) were acquired at rest. Adenosine (140 µg/kg/min) was then infused for at least 3 min, and the same three BOLD images were acquired during stress. Subsequently (4–5 min after commencing adenosine), a 0.03 mmol/kg bolus of gadolinium-based contrast (Gadoterate meglumine, Dotarem, Guerbet LLC, France) was injected, followed by 15 mL of normal saline at a rate of 6 mL/s for first-pass perfusion imaging. Adenosine was then discontinued and, after at least 20 min, another 0.03 mmol/kg bolus of gadolinium was given for post-adenosine rest perfusion imaging. Heart rates and BPs were recorded at baseline and at 1 min intervals during stress.

For late gadolinium enhancement (LGE) CMR, a top-up bolus of 0.09 mmol/kg of Gadoterate meglumine was administered immediately after rest perfusion imaging (a total dose of gadolinium of 0.15 mmol/kg). LGE images were acquired as described previously.^27^

### CMR data analysis

Left ventricular (LV) volumes, ejection fraction, and mass were calculated using cmr42^©^ (Circle Cardiovascular Imaging Inc., Calgary, Canada) by manually tracing the endocardial and epicardial contours in end-diastolic and end-systolic images, as described previously.^24^

Post-processing analysis of tagging images was performed using CIM-Tag software (Auckland, New Zealand). The peak systolic circumferential strain, global longitudinal strain, and diastolic strain rate data were analysed from the mid-short axis and horizontal long-axis tagging images, as described previously.^28^

The oxygenation-sensitive analysis technique has been described previously.^29^ Briefly, myocardial SI was measured after tracing endocardial and epicardial contours. Mean SIs were calculated for resting and stress conditions by averaging signal measurements from images during adenosine resting and stress, respectively, and were corrected for variations in heart rate, as described previously.^29^

For analysis of myocardial perfusion, SI over time curves was generated by tracing endocardial and epicardial contours (cmr42) after correction for displacement during breathing. A region of interest was drawn in the LV blood pool to obtain an arterial input function. Similar to oxygenation analysis, the myocardium was divided into equiangular segments on the basis of the American Heart Association segmentation model. Post-adenosine rest and stress myocardial perfusion upslopes were calculated using a five-point linear fit model of SI vs. time and normalized to the LV blood pool upslope. Myocardial perfusion reserve index (MPRI) was derived for each of the 16 segments, defined as the ratio of stress to rest normalized myocardial perfusion upslope^30^ in a blinded fashion by two operators (E.L. and A.L.).

For LGE analysis, areas of contrast enhancement were visually scored as absent or present by consensus of two experienced operators (E.L. and M.M.). LGE was considered present only if myocardial enhancement was confirmed on both SA and matching long-axis locations.

### ^31^P-MRS protocol

^31^P-MRS was performed to obtain the rest and exercise PCr/ATP from a voxel placed in the mid-ventricular septum, with the subjects lying prone with their heart over the centre of the ^31^P heart/liver coil in the iso-centre of the magnet, as described previously.^31,32^ Acquisition time was 9 min during rest and 9 min during leg exercise lying prone, with 2.5 kg weights attached to both ankles.

The rate pressure product (RPP) was calculated using the product of the heart rate and systolic BP, providing a measure of cardiac work. The starting RPP was calculated during the baseline spectral acquisition. Subjects then initiated exercise with repeated and alternate knee flexion, aiming to double the baseline RPP, with feedback given throughout. When maintained at a steady level of exercise, reached after 1 min, the exercise scans were acquired. Haemodynamic measurements were taken and recorded every minute and the mean exercise RPP calculated. Subjects maintained a steady exercise level during the 9 min acquisition of spectra. The volunteers stopped exercising on completion of the exercise spectrum. ^31^P-MRS post-processing analysis was performed as previously described.^33,34^

### Statistical analysis

All data are expressed as mean ± standard deviations, apart from diabetes duration which is expressed as median, and were checked for normality using the Kolmogorov–Smirnov test. Comparisons between the two groups were performed by Student's *t*-test. The χ^2^ test or Fisher's exact test was used to compare discrete data as appropriate. Bivariate correlations were performed using Pearson's or Spearman's method, as appropriate. Comparisons between rest and exercise energetics in patients and controls were performed with the two-tailed paired *t*-test. A *P*-value less than 0.05 was considered significant. All statistical analyses were performed with IBM SPSS Statistics version 20 (IBM, Armonk, NY, USA).

*A priori* sample size calculation was performed to detect a 13% drop in the PCr/ATP ratio in the T2DM cohort during stress. Based on pilot data (PCr/ATP rest 1.91 ± 0.25 and stress 1.65 ± 0.28) assuming two-tailed paired *t*-test analysis (*α* = 0.05 and *β* = 0.8), calculations suggested that 11 T2DM participants would be needed. A second *a priori* sample size calculation was also performed to detect a 10% difference in the PCr/ATP ratio in T2DM when compared with normal. Assuming two-tailed independent *t*-test analysis (*α* = 0.05 and *β* = 0.8), pilot data (PCr/ATP T2DM 1.74 ± 0.24 and normal populations 2.12 ± 0.26) suggested that eight T2DM and eight normal subjects would be needed to detect an 18% difference in the PCr/ATP ratio at rest. These targets were achieved in our study.

## Results

### Participant characteristics

Of the 39 diabetic patients screened in the study, 8 were excluded (main reasons: significant obstructive CAD on CCTA, systolic BP on screening >140 mmHg, and T wave inversions on ECG). Thirty-one patients (17 male, mean age 55 ± 9 years; BMI 28.7 ± 5.6 kg/m^2^) with T2DM, median diabetes duration 7 years [interquartile range (IQR): 1–8] and mean glycated haemoglobin level 7.4 ± 1.3%, and 17 controls (9 male, mean age 50 ± 14 years; BMI 27.1 ± 5.0 kg/m^2^) were studied.

Demographic, clinical, biochemical, and echocardiographic data are shown in *Table 1*. There were no significant differences in age, gender, systolic BP, and BMI between diabetic patients and controls. Diastolic BP and resting heart rate were statistically higher in the diabetic cohort, although remained within the normal range. A significant proportion of diabetics (77%) was on statin therapy; hence, total and low-density lipoprotein (LDL) cholesterol levels were lower than those in controls.

**Table 1:** Baseline characteristics of the study cohort

Variable	Controls, *N* = 17	Type 2 DM patients, *N* = 31	*P*-value
Age (years)	50 ± 14	55 ± 9	0.102
BMI (kg/m^2^)	27.1 ± 5.0	28.7 ± 5.6	0.302
Male (%)	53	58	0.739
Diabetes duration (years)	–	7 (IQR: 1–8)	
Systolic blood pressure (mmHg)	121 ± 12	127 ± 14	0.135
Diastolic blood pressure (mmHg)	69 ± 9	77 ± 8	0.007
Rest heart rate (b.p.m.)	60 ± 13	69 ± 9	0.036
Plasma fasting glucose (mmol/L)	4.9 ± 0.3	9.1 ± 3.2	<0.001
Glycated haemoglobin (%)	–	7.4 ± 1.3	
Glycated haemoglobin (mmol/mol)	–	60 ± 15	
Insulin (pmol/L)	–	135 ± 131	
Plasma triglycerides (mmol/L)	1.46 ± 0.7	1.47 ± 0.8	0.986
Plasma free fatty acids (mmol/L)	0.36 ± 0.20	0.60 ± 0.31	0.007
Total cholesterol (mmol/L)	5.2 ± 0.9	3.9 ± 0.8	<0.001
HDL (mmol/L)	1.36 ± 0.4	1.22 ± 0.4	0.273
LDL (mmol/L)	3.16 ± 0.6	1.9 ± 0.6	<0.001
Medications, *n* (%)			
Metformin	–	31 (97)	
Sulphonylurea	–	21 (68)	
Aspirin	–	11 (35)	
Statin	–	24 (77)	
ACE-I	–	21 (68)	

Values are mean ± standard deviations or percentages.

T2DM, type 2 diabetes mellitus; BMI, body mass index; HDL, high-density lipoprotein; LDL, low-density lipoprotein; ACE-I, angiotensin-converting enzyme inhibitors.

### Myocardial structure and systolic function

CMR results for LV volumes and function are summarized in *Table 2*. There was no significant difference in LV ejection fraction between patients with T2DM and controls. Diabetes was associated with concentric LV remodelling (LV mass: volume ratio T2DM, 0.98 ± 0.21 vs. controls, 0.70 ± 0.12; *P* < 0.001), with reduced LV diastolic volumes (*P* < 0.001) and increased maximal wall thickness (*P* = 0.016). LV mass did not differ between the two groups. Mid-ventricular systolic circumferential strain and global longitudinal strain were impaired in patients with T2DM compared with controls, indicating subtle alteration of both circumferential and longitudinal LV contractile function, in line with a previous study.^35^

**Table 2:** CMR results in patients vs. controls

Variable	Controls, *N* = 17	Type 2 DM patients, *N* = 31	*P*-value
LV end-diastolic volumes (mL)	161 ± 39	125 ± 30	0.001
LV end-systolic volumes (mL)	48 ± 16	40 ± 18	0.137
LV stroke volume (mL)	105 ± 25	88 ± 25	0.022
LV ejection fraction (%)	70 ± 5	69 ± 9	0.535
LV mass index (g/m^2^)	52 ± 14	60 ± 13	0.056
LV mass (g)	109 ± 30	121 ± 31	0.235
LV diastolic wall thickness (mm)	9.3 ± 1.2	10.6 ± 1.8	0.016
LV mid-ventricular circumferential systolic strain (%)	−(19 ± 3)	−(14 ± 2)	<0.001
LV mass/end-diastolic volume (g/mL)	0.70 ± 0.12	0.98 ± 0.21	<0.001
LV mid-ventricular diastolic strain rate (s^−1^)	65 ± 13	62 ± 26	0.749
LV global longitudinal strain (%)	−(11.4 ± 2.8)	−(9.6 ± 2.9)	0.049

Values are mean ± standard deviations or percentages.

T2DM, type 2 diabetes mellitus; CMR, cardiac magnetic resonance; LV, left ventricle.

### Haemodynamic measurements

Rest, post-adenosine rest, physiological stress and pharmacological stress BP, and heart rate responses are summarized in *Table 3*. Adenosine stress and exercise led to similar percentage increases in RPP.

**Table 3:** Haemodynamic measurements

Variable	Controls	T2DM	*P*-value
^31^P-MRS exercise stress			
Rest heart rate (b.p.m.)	55 ± 10	69 ± 8	<0.001
Stress heart rate (b.p.m.)	78 ± 10	84 ± 10	0.076
Rest blood pressure (mmHg)	121 ± 12	127 ± 14	0.135
Stress blood pressure (mmHg)	126 ± 15	147 ± 20	0.002
Rest RPP (b.p.m.×mmHg)	6832 ± 1441	8766 ± 1318	<0.001
Stress RPP (b.p.m.×mmHg)	9926 ± 1761	12 264 ± 2204	0.002
Increase in RPP (%)	48 ± 30	41 ± 22	0.381
Adenosine stress CMR			
Post-adenosine rest heart rate (b.p.m.)	60 ± 13	69 ± 9	0.036
Stress heart rate (b.p.m.)	77 ± 16	85 ± 9	0.054
Stress blood pressure (mmHg)	121 ± 9	130 ± 15	0.075
Post-adenosine rest RPP (b.p.m.×mmHg)	6982 ± 1494	9382 ± 2106	0.001
Stress RPP (b.p.m.×mmHg)	10 048 ± 2856	12 479 ± 2819	0.014
Increase in RPP (%)	44 ± 26	35 ± 29	0.369

Values are mean ± standard deviations or percentages.

T2DM, type 2 diabetes mellitus; CMR, cardiac magnetic resonance; b.p.m., beats per minute; BP, blood pressure; RPP, rate pressure product.

### Changes in rest and exercise myocardial energetics

**Figure 2:**
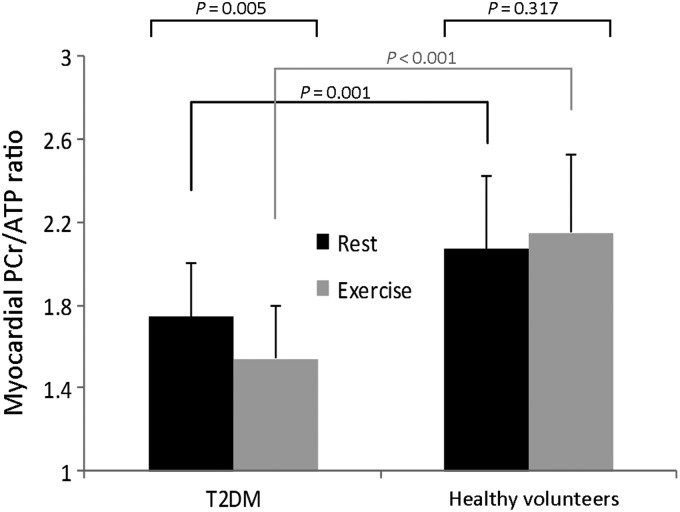
Column graphs with means and standard deviations showing differences in rest and exercise myocardial PCr/ATP ratios between controls and patients with T2DM. Bars show mean PCr/ATP ratios and error bars indicate standard deviations.

**Figure 3:**
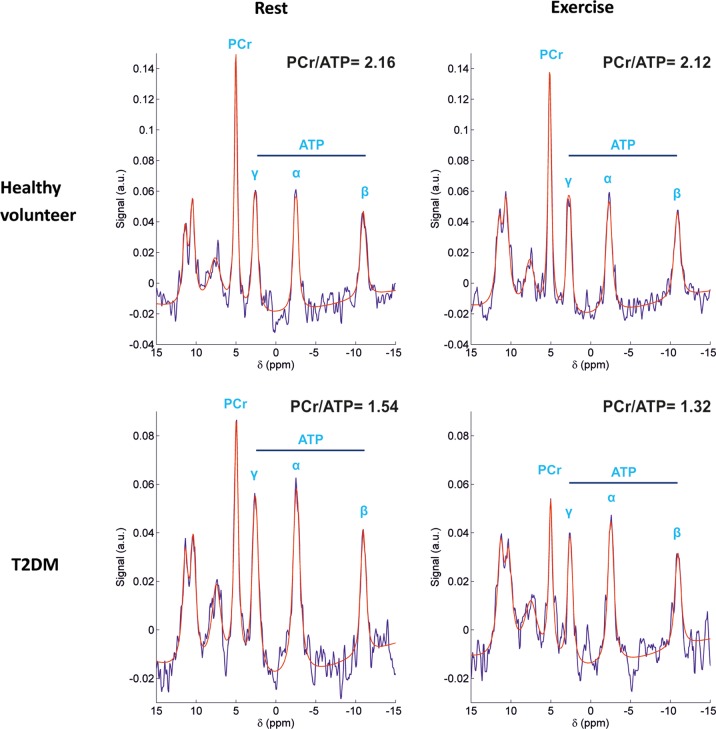
Representative rest and exercise ^31^P-MR spectra examples. Rest and exercise myocardial phosphorus spectra in a healthy volunteer (top row) and a patient with T2DM. Note a further decrease in already lower rest PCr/ATP in the patient with T2DM during exercise.

Diabetes was associated with a 17% decrease in PCr/ATP at rest compared with controls (*P* = 0.001), and there was a further 12% decrease in PCr/ATP with exercise (mean rest PCr/ATP 1.74 ± 0.26 to mean exercise PCr/ATP 1.54 ± 0.26; *P* = 0.005; *Figure 2*). In contrast, there was no significant change in PCr/ATP in healthy controls with exercise. *Figure 3* shows the representative rest and exercise ^31^P-MR spectra.

### Changes in myocardial perfusion and oxygenation under adenosine stress

**Figure 4:**
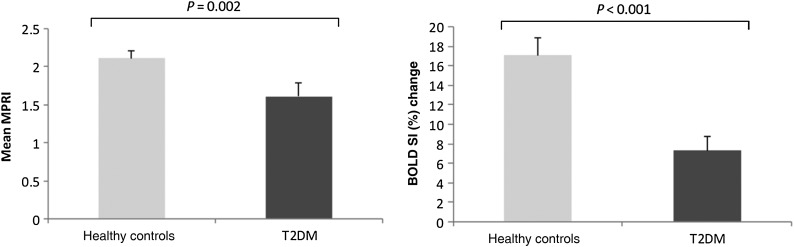
Column graphs with means and standard deviations showing differences in MPRI and BOLD SI (%) change between controls and patients with T2DM. Bars show mean PCr/ATP ratios and error bars indicate standard deviations.

**Figure 5:**
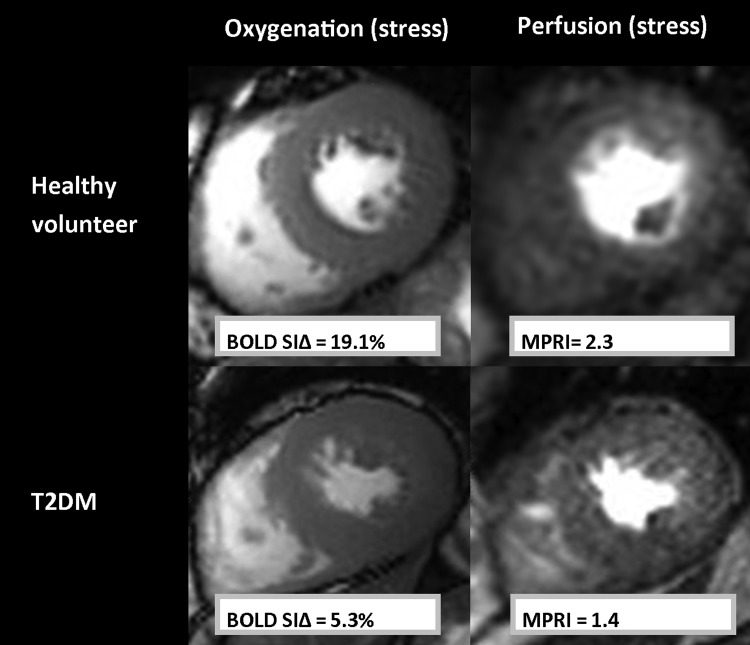
Representative CMR perfusion and oxygenation examples. Oxygenation and corresponding perfusion images in a healthy volunteer (top row) and a patient with T2DM. Perfusion reserve (mean MPRI) and oxygenation SIΔ (BOLD SIΔ) were impaired in the patient.

Mean MPRI in the T2DM group was 24% lower than that in controls (*P* = 0.002; *Figure 4*). During vasodilator stress, patients with T2DM showed evidence of blunted oxygenation response [signal intensity change (SIΔ): T2DM 7.3 ± 7.8%], compared with controls (SIΔ: 17.1 ± 7.2%, *P* < 0.001; *Figure 4*). *Figure 5* shows representative CMR images of oxygenation and perfusion.

### Assessment of myocardial scarring using LGE imaging

No areas of myocardial enhancement indicative of replacement or interstitial fibrosis were identified in either diabetic patients or normal controls.

### Correlations among myocardial oxygenation, perfusion, energetics, and strain

In line with a previous study,^3^ we found that MPRI had no significant correlation with PCr/ATP at rest. However, a positive correlation with PCr/ATP was obtained during exercise (*r* = 0.50, *P* = 0.001). Impaired MPRI was associated with blunting of myocardial oxygenation during vasodilator stress (*r* = 0.40, *P* = 0.023). There was also a positive correlation between exercise PCr/ATP and oxygenation SIΔ (*r* = 0.32, *P* = 0.025), whereas there was no correlation between the rest PCr/ATP and oxygenation SIΔ. Systolic circumferential strain, which is a CMR marker that is known to represent LV contractile function, correlated with rest PCr/ATP (*r* = 0.40, *P* = 0.036) and exercise PCr/ATP (*r* = 0.50, *P* = 0.003).

## Discussion

Using CMR and ^31^P-MRS to study patients with T2DM free of significant obstructive epicardial CAD, we assessed the effects of diabetes on cardiac metabolic reserve and how metabolic reserve relates to both myocardial oxygenation and perfusion reserve. We demonstrated that during exercise, the pre-existing energetic deficit in patients with diabetes, as determined by PCr/ATP, is exacerbated. We confirm the previous finding of an impaired myocardial perfusion reserve in diabetes.^3,36^ We now also show that patients with diabetes not only have impaired perfusion, but also evidence of blunted myocardial oxygenation at stress. Finally, we demonstrate that although myocardial energy metabolism at rest does not correlate with coronary microvascular dysfunction and is primarily a result of an intrinsic metabolic deficit, during exercise microvascular dysfunction exacerbates the energetic deficit.

### Diabetes and cardiac metabolic reserve

Myocardial energetic compromise, indicated by decreased PCr/ATP, is a predictor of mortality,^11^ linked to contractile dysfunction,^9,11^ and is a well-recognized complication of diabetes.^3,4^ Here, we demonstrate exacerbation of this energetic deficit during exercise in stable patients with diabetes, indicating impaired cardiac metabolic reserve.

The healthy myocardium has rapid response mechanisms to deal with acute changes in energy demand, providing a large metabolic reserve.^37^ These mechanisms include increased contribution of carbohydrates to energy production glycogenolysis,^38^ increased glucose uptake and glycolysis,^39^ and increased rates of phosphotransferase reactions.^40^ The primary energy reserve compound in the heart is PCr, and the enzyme creatine kinase is thought to allow the transfer of the high-energy phosphate bond between ATP and PCr, through the phosphotransferase reactions, in order to diffuse energy from the mitochondria to the myofibrils as PCr.^9^ These changes require the metabolic machinery to be flexible when, in contrast, diabetes is associated with metabolic inflexibility. The further drop in PCr/ATP during exercise in our patients with diabetes can potentially be explained by metabolic inflexibility,^41^ insufficient oxygen delivery, in addition to an impaired oxidative metabolism in diabetes resulting in reduced ATP production.^7^

The causal role of altered energetics in contractile dysfunction in diabetic hearts is controversial. In our study, we show a correlation between myocardial systolic strain and the rest and stress PCr/ATP, suggesting a link between the two; however, the causality of this relationship will need to be investigated in future studies.

Given the fact that we have shown significant abnormalities in metabolic reserve, myocardial perfusion reserve, and myocardial oxygenation response to adenosine stress in a stable diabetes population, similar or amplified findings could potentially be expected in diabetic patients with more advanced cardiovascular disease. Future studies should confirm this.

### Myocardial tissue perfusion and oxygenation in diabetic cardiomyopathy

Impaired myocardial perfusion, either due to coronary microvascular dysfunction or due to endothelial dysfunction in diabetes, results in a failure to increment myocardial blood flow during acute increases in cardiac workload.^3,5,36,42^ Coronary microvascular dysfunction in diabetes is a multifactorial phenomenon, related to changes in perivascular and interstitial fibrosis,^43^ reduced capillary density, and autonomic neuropathy.^44^ Myocardial hypoperfusion does not always reflect tissue ischaemia, as oxygen demand may vary in different pathophysiological states. Indeed, some degree of dissociation exists between flow and oxygenation in the setting of CAD and dilated cardiomyopathy.^29,45^

The interplay between myocardial perfusion and oxygenation in diabetes has previously not been explored. Our study shows that, in the context of well-controlled diabetes, myocardial perfusion reserve is impaired and oxygenation during vasodilator stress is blunted and that these changes correlate with each other. These findings support the concept that hypoperfusion as a result of microvascular dysfunction plays a role in the impaired ability to increase and/or maintain myocardial oxygenation during vasodilatory stress in diabetes.

The endothelium has been recognized to be a major regulator of vascular tone and growth. Experimental and clinical studies have demonstrated the association between diabetes and endothelium-dependent relaxation impairment. Although in our study we assessed myocardial perfusion reserve to adenosine stress, this parameter is both endothelium- and non-endothelium-dependent, and an observational study such as ours cannot clearly identify the mechanisms responsible for the reduced coronary vasodilation response to adenosine.

## Study limitations

This study is limited by a relatively small sample size, in line with its proof-of-principle nature, and further studies are needed to understand the complex interaction between metabolic reserve and other factors. The principal limitation of our study is the lack of repeated assessment of myocardial function during stress. However, previous studies have shown exaggerated diastolic and systolic dysfunction in response to stress in patients with diabetes.^46,47^ Subjecting our participants to a third stress protocol (in addition to leg exercise during the acquisition of ^31^P-MRS and adenosine stress for the assessment of MPRI and oxygenation SIΔ) was deemed too high a burden on study subjects as this would lead to significantly longer adenosine infusion times, higher risk of adverse event rates, and high drop-out rates. For the same reasons, we have not carried out invasive coronary angiography for the assessment of endothelium-dependent coronary vasodilatation and vascular smooth muscle cell responsiveness. Although the impaired myocardial perfusion reserve demonstrated is commonly attributed to microvascular disease, in the current study, we cannot mechanistically differentiate between endothelial dysfunction and impaired smooth muscle relaxation as potential causes for the observed changes in diabetes.

The leg flexion stress was submaximal during the 9 min of acute physical exercise, with an average RPP increase of 40–50% in patients and controls, likely representing the physical constraints of exercising in an MRI scanner. However, this moderate exercise reflects typical levels of exercise that patients with diabetes would perform in daily life. Although mean rest and exercise RPP were higher in diabetics, increases in RPP were similar in the two groups.

CCTA was not performed in the normal volunteers to prevent unnecessary ionizing radiation exposure. Significant CAD was deemed to be unlikely in this normal cohort, and this is further supported by the fact that perfusion and oxygenation values were within the normal range.

## Clinical implications

The current study provides important insights into the interplay of perfusion, oxygenation, and metabolic changes during stress in the diabetic heart. We have identified the presence of markers of poor prognosis such as myocardial energetic compromise and impaired perfusion reserve,^9,11^ which have been linked to contractile dysfunction and are predictors of mortality.^9,11,48^ Moreover, these findings were detected in a subclinical setting of well-controlled and stable patients, and more profound alterations may be expected in overt diabetic cardiomyopathy.

Our findings suggest that strategies aimed at improving metabolic reserve and myocardial oxygenation together, such as pharmacological activation of the hypoxia-inducible factor pathway, which increases angiogenesis and oxygen-carrying capacity and metabolically upregulates oxygen-independent ATP synthesis,^49^ may in the future become therapeutic targets for patients with diabetic cardiomyopathy.

Future proof-of-principle clinical studies may use stress myocardial PCr/ATP and the BOLD SIΔ to monitor the early energetic and vascular response of the heart to novel therapies, and it is possible that these methods may provide surrogate markers of long-term prognostic effects.

## Conclusions

The pre-existing energetic deficit in diabetic cardiomyopathy is further exacerbated during exercise. Although the myocardial PCr/ATP ratio at rest is not related to coronary microvascular dysfunction and is primarily a result of intrinsic metabolic dysfunction, during exercise, microvascular dysfunction appears to exacerbate the energetic deficit. Diabetes is associated with a reduction in perfusion reserve severe enough to lead to myocardial deoxygenation and further exacerbation of the energetic abnormalities during increased workload. These mechanisms may contribute to the pathophysiology of the cardiomyopathy process in diabetes.

## Funding

The study was supported by the Oxford Partnership Comprehensive Biomedical Research Centre with funding from the Department of Health's National Institute for Health Research Biomedical Research Centers. S.N. acknowledges support from the Oxford British Heart Foundation Center of Research Excellence. C.T.R. is supported by a Sir Henry Dale Fellowship jointly funded by the Wellcome Trust and the Royal Society (Grant no. 098436/Z/12/Z). Funding to pay the Open Access publication charges for this article was provided by Wellcome Trust (Grant Number 098436/Z/12/Z).

**Conflict of interest:** none declared.
